# Variants identified in *PTK7* associated with neural tube defects

**DOI:** 10.1002/mgg3.584

**Published:** 2019-01-28

**Authors:** Yunping Lei, Sung‐Eun Kim, Zhongzhong Chen, Xuanye Cao, Huiping Zhu, Wei Yang, Gary M. Shaw, Yufang Zheng, Ting Zhang, Hong‐Yan Wang, Richard H. Finnell

**Affiliations:** ^1^ Department of Nutritional Sciences Dell Pediatric Research Institute, University of Texas at Austin Dell Medical School Austin Texas; ^2^ Obstetrics and Gynecology Hospital, State Key Laboratory of Genetic Engineering at School of Life Sciences, Institute of Reproduction and Development Fudan University Shanghai China; ^3^ Departments of Molecular and Cellular Biology and Medicine Baylor College of Medicine Houston Texas; ^4^ Department of Pediatrics, Division of Neonatology Stanford University School of Medicine Stanford California; ^5^ Beijing Municipal Key Laboratory of Child Development and Nutriomics Capital Institute of Pediatrics Beijing China; ^6^ Collaborative Innovation Center for Genetics & Development, School of Life Sciences Fudan University Shanghai China; ^7^ Present address: Center for Precision Environmental Health, Departments of Molecular and Cellular Biology and Medicine Baylor College of Medicine Houston Texas 77030; ^8^ Present address: Asuragen Inc. 2150 Woodward St #100 Austin TX 78744

**Keywords:** neural tube defects, planar cell polarity, *PTK7*

## Abstract

**Background:**

Variants in planar cell polarity (PCP) pathway genes have been repeatedly implicated in the pathogenesis of NTDs in both mouse models and in human cohorts. Mouse models indicate that the homogenous disruption of the *Ptk7* gene, a PCP regulator, results in craniorachischisis; while embryos that are doubly heterozygous for *Ptk7^XST87^* and *Vangl2* ^Lp ^mutations present with spina bifida.

**Methods:**

In this study, we initially sequenced exons of the human *PTK7* gene in 192 spina bifida patients and 190 controls from a California population. A phase II validation study was performed in 343 Chinese NTD cohort. Functional assays including immunoblotting and immunoprecipitation were used to study identified variants effect on PTK7 function.

**Results:**

We identified three rare (MAF <0.001) missense heterozygous *PTK7* variants (NM_001270398.1:c.581C>T, p.Arg630Ser and p.Tyr725Phe) in the spina bifida patients. In our functional analyses, p.Arg630Ser affected PTK7 mutant protein stability and increased interaction with Dvl2, while the p.Thr186Met variant decreased PTK7 interactions with Dvl2. No novel predicted‐to‐be‐damaging variant or function‐disrupted *PTK7* variant was identified among the control subjects. We subsequently re‐sequenced the *PTK7* CDS region in 343 NTDs from China to validate the association between *PTK7* and NTDs. The frequency of *PTK7* rare missense variants in the Chinese NTD samples is significantly higher than in gnomAD controls.

**Conclusion:**

Our study suggests that rare missense variants in *PTK7* contribute to the genetic risk of NTDs.

## 1 INTRODUCTION

Neural tube defects (NTDs; OMIM#182940) are a group of congenital malformations that affect the brain and spinal cord. NTDs are known to occur in approximately 0.69–2.19 of every 1,000 newborns, with varying prevalence across different populations and different geographical regions (Botto, Moore, Khoury, & Erickson, 1999; Zaganjor et al., 2016). The most common NTD observed at birth is spina bifida, which results from a failure of fusion of the neural folds in the spinal region below the level of T12 (Rossi et al., 2004). The etiology of NTDs is complex and involves both genetic and environmental factors (Kibar, Capra, & Gros, 2007). Although peri‐conceptional supplementation with folic acid can reduce the frequencies of NTDs by up to 70% (Berry et al., 1999; Czeizel & Dudas, 1992; MRC VITAMIN STUDY RESEARCH GROUP, 1991), the mechanism by which folate benefits the developing embryo to prevent NTDs remains unclear. More than 300 genes have been found to be associated with NTDs in mice, but progress in delineating the molecular basis of human NTDs has been extremely limited (Harris & Juriloff, 2010; Wilde, Petersen, & Niswander, 2014). Over the past decade, several planar cell polarity (PCP) genes were found to be associated with an increased risk for NTDs in humans, including: *VANGL1* (Kibar et al., 2009; Kibar, Torban et al., 2007), *VANGL2* (Kibar et al., 2011; Lei et al., 2010), *PRICKLE1* (Bosoi et al., 2011), *FZD6* (De Marco et al., 2012), *CELSR1* (Allache, De Marco, Merello, Capra, & Kibar, 2012; Lei et al., 2014; Robinson et al., 2012), *SCRIB* (Lei et al., 2013) and *LRP6* (Allache et al., 2014; Lei et al., 2015). A PCP effector gene *FUZ* (Seo et al., 2011), and a PCP regulator gene *DACT1* (Shi et al., 2012), have also been found to be associated with human NTDs. A recent comprehensive genetic analysis targeting PCP genes revealed that all of the CELSR family members contribute to the etiology of human NTDs (Chen, Lei, Cao et al., 2018). Other PCP genes, and PCP effector and regulator genes also play a potential role in convergent extension movements and neural tube closure (NTC), and therefore need to be further studied in human NTD cohorts.

Protein tyrosine kinase 7 *(PTK7*, OMIM#: 601890) is a regulator of PCP in vertebrate embryos (Lu et al., 2004). It is required for a broad range of morphogenetic processes regulated by genes within the PCP signaling pathway. One such process is convergent extension, which describes a morphogenetic pattern of cell movement required for proper NTC. Defects in convergent extension are considered to be a hallmark of a malfunctioning PCP signaling pathway (Roszko, Sawada, & Solnica‐Krezel, 2009). *PTK7* codes for a one‐pass transmembrane protein with tyrosine kinase homology. It can act as a Wnt co‐receptor to activate the PCP pathway and inhibit canonical Wnt signaling (Peradziryi, Tolwinski, & Borchers, 2012). Previous studies demonstrated that *PTK7* is required for convergent extension and cell movements in *Xenopus*, zebrafish and in mice (Golubkov et al., 2010; Wehner, Shnitsar, Urlaub, & Borchers, 2011; Yen et al., 2009). PTK7 missense variants were identified in an NTD cohort collected from Italy and Canada (Wang et al., 2015). However, its association with NTDs in other populations is unclear, which prompted us to investigate the *PTK7* as a risk factor for human NTDs in a US NTD cohort and a Chinese NTD cohort.

## 2 MATERIALS AND METHODS

### 2.1 Ethical compliance

This study was approved by IRB Committee at the University of Texas at Austin IRB (approve #: 2010‐09‐0043 and 2010‐09‐0057). All US samples were obtained with approval from the State of California Health and Welfare Agency Committee for the Protection of Human Subjects. All Chinese samples were obtained with approval from the institutional review board of Fudan University and Capital Institute of Pediatrics, Beijing, China. Consent forms were signed by all of the parents of participating minors.

### 2.2 Human subjects

Samples were obtained from a case–control study conducted by the California Birth Defects Monitoring Program (CBDMP). The CBDMP is an active, population‐based surveillance system for collecting information on infants and fetuses with congenital malformations, which has been described elsewhere (Croen, Shaw, Jensvold, & Harris, 1991). Included in this study were 192 isolated infants with spina bifida (cases) and 190 non‐malformed infants (controls) as previously reported (Lei et al., 2014).

Cases were randomly selected from all live born cases and a random sample of non‐malformed control infants ascertained by the CBDMP corresponding to birth years 1983–1999. The case and control infants were linked to their newborn bloodspot.

The Chinese NTD samples that were utilized for the validation studies were obtained from either aborted fetuses (22.6 ± 7.0 weeks) or children with spina bifida (5.5 ± 3.9 years) (Table S1), which was also described elsewhere (Chen, Kuang, Finnell, & Wang, 2018; Chen, Lei, Cao et al., 2018; Qiao et al., 2016).

### 2.3 DNA resequencing of *PTK7 *and *VANGL2*

DNA was extracted from newborn screening blood spots using the Gentra Puregene DNA Extraction Kit (Qiagen, Valencia, CA). The genomic structure of human *PTK7* was determined using the NCBI GenBank (NT_007592.15, NM_001270398/ENST00000481273.5 and NP_001257327). The 20 exons of *PTK7* were amplified by polymerase chain reactions (PCR) using primers flanking exon‐intron junctions. Primer sequences are available upon request. The PCR products were sequenced using the Prism Bigdye Terminator Kit (v3) on an ABI 3730XL DNA analyzer (Life Technologies, Carlsbad, CA). Both case and control samples were sequenced with either a specific forward or reverse primer. The detected variants were confirmed by repeating the PCR and re‐sequencing from both directions. *VANGL2* was re‐sequenced in the 192 NTD cases following our previous publication (Lei et al., 2010). Sequencing results were analyzed using the Mutation Surveyor software V4.0.5 (Softgenetics, Stage College, PA).

### 2.4 Immunocytochemistry

The tGFP‐PTK7 plasmid was purchased from Origene (CAT#: RG209690). PTK7 variants were introduced into tGFP‐PTK7 by site‐direct mutagenesis using GeneArt® Site‐Directed Mutagenesis System (Thermo Fisher Scientific, CAT#:A14604). Hela cells were plated on the cover glass and were transfected with tGFP‐PTK7 (WT and R630S) using Lipofectamine LTX (Invitrogen) according to the manufacturer's instruction. Cells were fixed with paraformaldehyde (PFA) for 30 min at 48 hr post transfection and were mounted with prolong mounting solution (Invitrogen). The cell images were obtained with a laser scanning confocal microscope (LSM710, Leica).

### 2.5 Western blot (WB) analysis

HEK293T cells were transfected with tGFP‐PTK7 (WT or mutant) using Lipofectamine 2000 (Invitrogen). The cells were lysed with radioimmunoprecipitation assay (RIPA) buffer at 48 hr post transfection. For Cyclohexamide (CHX) treatment, the cells were treated with CHX (1 g/ml) for 24 hr before lysis. The lysates were immunoblotted with anti‐tGFP (Origene) or anti‐GAPDH (Cell signaling), 1RDye® 800CW goat anti‐rabbit IgG secondary antibodies (LI‐COR), and 1RDye® 680CW goat anti‐mouse IgG secondary antibodies (LI‐COR). The images were captured by Odyssey® (LI‐COR). The statistical analysis was performed using a student *t* test on data obtained from three independent experiments.

### 2.6 Immunoprecipitation

HEK293T cells were transfected with tGFP‐PTK7 (WT or variants) and HA‐Dvl2 or HA‐Vangl2 with Lipofectamine 2000. The cells were lysed with RIPA buffer at 48 hr post transfection. The lysates were immunoprecipitated with anti‐HA antibody (Santa Cruz Biotechnology) along with protein G bead (Pierce) for overnight at 4°C. The immunoprecipitated lysates were washed three times with RIPA buffer and were further analyzed by immunoblotting with anti‐tGFP or anti‐HA.

### 2.7 Bioinformatics

Variants were annotated according to the HGVS nomenclature (http://www.hgvs.org/mutnomen/). Nucleotide numbering reflects cDNA numbering with +1 corresponding to the A of the ATG translation initiation codon 1 in the reference sequence, according to the journal guidelines. A variant was designated as novel if it was not found in either ExAC or gnomAD databases. The potential pathogenic effect of the missense variants on protein function was predicted using two online programs: *PolyPhen* (Polymorphism Phenotyping) (http://genetics.bwh.harvard.edu/pph/) and *PANTHER* (Protein Analysis Through Evolutionary Relationships) (http://www.pantherdb.org/). Multiple alignments of the PTK7 proteins were performed using the *CLUSTAL W *program built in *Mega software* (V5.1), available online (http://www.megasoftware.net/). Localization of the variants in protein domains was assessed by *Uniprot* (http://www.uniprot.org/). Gene scheme structure was generated by using *Lollipops *(Jay & Brouwer, 2016).

### 2.8 Association analysis

Rare variants were defined as those having a minor allele frequency (MAF) of no more than 1%. Variants with a frequency greater than 0.01 were considered to be common (MAF ≥0.01) variants. Fisher's exact test (two‐tailed) was used to test for association with rare variants; while the Chi‐square test statistic was used to assess association with common variants. *p* < 0.05 was considered as significant.

## 3 RESULTS

### 3.1 Sequencing analyses of *PTK7* in spina bifida

DNA resequencing of all 382 subjects (192 spina bifida cases and 190 non‐malformed controls) identified 9 rare missense *PTK7* DNA variants (MAF <1%) and five common variants (MAF ≥1%). Amongst the 192 spina bifida cases, we detected three rare missense variants; p.Thr186Met (c.557C>T), p.Arg630Ser (c.1888C>A), and p.Tyr725Phe (c.2174A>T). These alleles were absent from all 190 non‐malformed controls. One of them (p.Arg630Ser) appears to be novel, as it was absent from both the *ExAC database* (http://exac.broadinstitute.org/) and gnomAD database (http://gnomad.broadinstitute.org/) (Table 1). We also identified three control‐specific rare missense variants that were not present in our spina bifida cases. All of the variants identified in controls except p.Ala856Thr (c.2566G>A) were found in the *ExAC database*. However, the p.Ala856Thr allele was predicted to be benign by both *SIFT* and *PolyPhen* software. Three rare missense variants and five common (MAF >1%) single nucleotide variants (SNVs) were detected in both cases and controls, although none of them had previously been associated with an increased risk for spina bifida (*p* > 0.05) (Table 2). VANGL2 gene in the 192 spina bifida cases was also sequenced, but no rare missense variant was identified.

**Table 1:** Novel and known rare missense variants (MAF <0.01) in the coding sequence of PTK7 gene detected in this study

Nucleotide change	rs ID	aa change	NTD(192)/Control(190)	PolyPhen prediction	SIFT prediction	Uniprot domain	ExAC counts(frequency)	gnomAD counts(frequency)
c.557C>T	rs767634504	p.Thr186Met	1/0	Benign	TOLERATED	Ig‐like C2‐type 2	7/122,018 (5.8e‐5)	19/276,314(6.876e‐5)
**c.1888C>A**	**NA**	**p.Arg630Ser**	**1/0**	**Possibly damaging**	**TOLERATED**	**Ig‐like C2‐type 7**	**ND**	**ND**
**c.2174A>T**	**NA**	**p.Tyr725Phe**	**1/0**	**Possibly damaging**	**TOLERATED**	**Transmembrane**	**ND**	**1/246,014(4.05e‐6)**
c.811C>T	rs763808505	p.Arg271Cys	0/1	Probably damaging	TOLERATED	Ig‐like C2‐type 3	2/122,736(1.7e‐5)	3/246,158(1.219e‐5)
c.881G>A	rs748100799	p.Arg294His	0/1	Probably damaging	DAMAGING	Ig‐like C2‐type 3	2/122,378 (1.7e‐5)	11/245,966(4.472e‐5)
c.2024C>T	rs79644111	p.Thr675Met	1/1	Probably damaging	TOLERATED	Ig‐like C2‐type 7	76/122,412(6.3e‐4)	215/277,052(7.76e‐‐4)
c.2236A>C	rs150631466	p.Met746Leu	2/1	Benign	TOLERATED		97/114,576(8.5e‐4)	241/274,626(8.776e‐4)
c.2566G>A	NA	p.Ala856Thr	0/1	Benign	TOLERATED	Protein kinase	ND	ND
c.3113G>A	rs34865794	p.Arg1038Gln	2/1	Benign	TOLERATED	Protein kinase	1676/122,412(1.4e‐2)	4,132/276,228(1.429e‐2)

Bold value indicates case specific variants which are predicted to be damaging by PolyPhen.

**Table 2:** Common variants (MAF ≥0.01) in the coding sequence of PTK7 gene detected in this study

Nucleotide change (NM_002821.3)	dbSNP ID	Amino acid change (NP_002812.2 )	MAF control/case	*p* value	gnomad MAF
c.1176C>T	rs56004029	p.His392His	0.018/0.023	0.814974135	0.01281
c.1228A>T	rs34021075	p.Thr410Ser	0.016/0.005	0.277069927	0.01218
c.1851G>A	rs6905948	p.Gly617Gly	0.374/0.382	0.88104387	0.3633
c.2235G>C	rs9472017	p.Glu745Asp	0.013/0.003	0.212242927	0.01155
c.2330C>T	rs34764696	p.Ala777Val	0.027/0.030	0.989891728	0.04793

**Figure 1:**
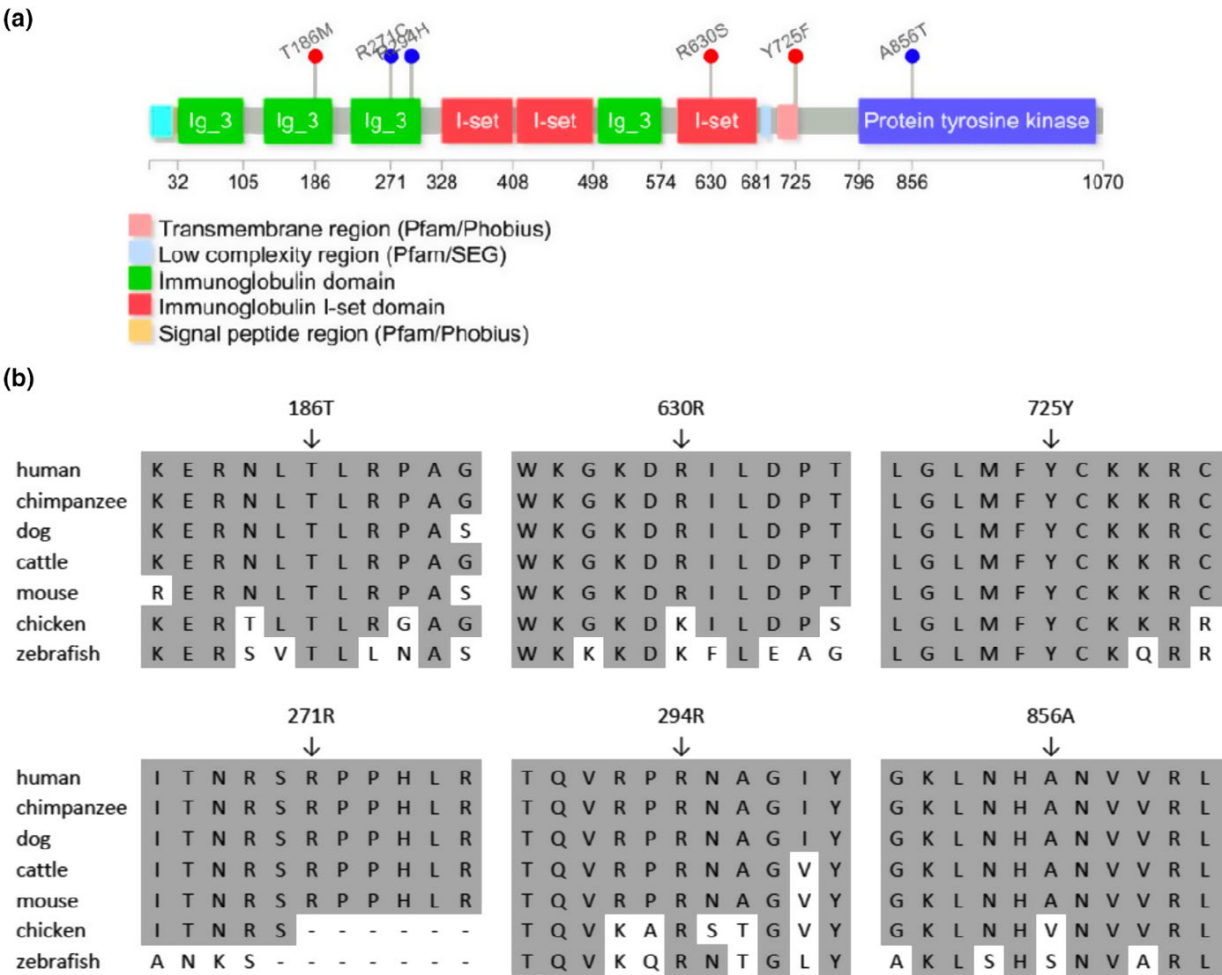
Identification of NTD‐associated non‐synonymous amino acid substitutions in the *PTK7* gene. (a) Conserved domains of the PTK7 and positions of the detected novel rare missense mutations. Red mutations in the panel were identified in NTD cases, blue variants were detected in controls. (b) Alignment of PTK7 ortholog protein sequences using the ClustalW method. Conserved residues were shaded by *GeneDoc*. The following sequences were used: human, NP_002812.2; chimpanzee, XP_518486.2; dog, XP_538929.2; cattle, NP_001179894.1; mouse, NP_780377.1; chicken, NP_001026206.1; and zebrafish, XP_0026673

The *PTK7* p.Thr186Met (c.557C>T) variant was found in one case infant but not in any of the 192 controls. The minor allele frequency of p.Thr186Met (c.557C>T) is 5.8 × 10^−5^ in ExAC database and 6.8 × 10^−5^ in gnomAD database. This variant changed a hydrophilic residue into a hydrophobic residue. The PTK7 186 methionine residue is located in a highly conserved region which belongs to the second immunoglobulin‐like domain of PTK7 (Figure 1). The p.Arg630Ser (c.1888C>A) was found in another case infant, but not in any controls. *PolyPhen* predicted that this variant is possibly pathogenic. Although this site was occupied by a lysine and not an arginine in chickens and zebrafish, it is still considered as a positively charged amino acid. In the spina bifida case, the arginine residue was changed to serine, which is a hydrophilic uncharged amino acid. The p.Tyr725Phe (c.2174A>T) was detected in another spina bifida case but not in any control. This variant affects a highly conserved residue, predicted to be localized at the trans‐membrane domain of PTK7. Tyrosine is a hydrophilic amino acid, while phenylalanine is a hydrophobic residue. *PolyPhen* software predicted that this p.Tyr725Phe variant would be damaging.

We extended our re‐sequencing analyses of *PTK7* to a second cohort of 343 aborted fetuses with NTD cases collected in China for association validation. Eleven predicted‐to‐be damaging rare variants were detected in NTDs (Table 3), and one common (MAF >1%) single nucleotide variant was identified in both cases and in Chinese controls from 1,000 Genomes Project (1KGP) (Table S2). However, it was not associated with an increased risk for NTDs (*p* = 0.92). In the Chinese NTD cohort, the frequency of rare (MAF <1%) *PTK7* missense DNA variants was 6.12% (21/343), while rare missense variants frequency in the Han Chinese population from 1KGP is 2.88% (6/208) and in the Genome Aggregation Database (gnomAD) is 3.30% (4,578/138,632) (Table 3). No significant difference (*p* = 1) was found in the *PTK7* rare missense variants frequency between Han Chinese in 1KGP and that of gnomAD. Thus, to increase power, gnomAD *PTK7* rare missense variants data were used as controls. *PTK7* rare variants are significantly (*p* = 0.00899) enriched in Han Chinese NTDs than in gnomAD controls.

**Table 3:** Rare missense variants (MAF <0.01) in the coding sequence of *PTK7* gene detected in 343 Chinese NTDs

chr6 position	rs#	DNA change	aa change	SIFT	PolyPhen	No. of carriers	Phenotype(num)^b^	MAF in gnomAD
43096973	rs757907657	c.362G>A	p.Arg121His	Tolerated	Benign	1	SB	3.56E‐05
43096994	rs780174508	c.383A>G	p.Asn128Ser	Deleterious	Probably damaging	1	EX	7.14E‐06
43098060	rs149112329	c.587G>A	p.Arg196Gln	Tolerated	Benign	1	EC	2.20E‐04
43100174	NA^a^	c.1001C>T	p.Pro334Leu	Tolerated	Benign	1	AE	NA
43109441	rs777499261	c.1678G>A	p.Ala560Thr	Tolerated	Possibly damaging	1	SB	2.39E‐05
43109699	rs762888862	c.1823G>A	p.Arg608His	Tolerated	Benign	1	SB	4.99E‐05
43111200	rs148120569	c.2117C>G	p.Pro706Arg	Deleterious	Probably damaging	5	AE(2), CRS, SB(2)	3.83E‐04
43112206	rs200622454	c.2293G>A	p.Gly765Arg	Deleterious	Probably damaging	2	SB	5.66E‐05
43112236	rs775883985	c.2323G>A	p.Val775Met	Deleterious	Benign	1	SB	4.77E‐05
43112282	rs746367585	c.2369G>T	p.Arg790Leu	Deleterious	Benign	4	SB(2),EC,AE	1.06E‐05
43112285	rs55820547	c.2372A>G	p.His791Arg	Tolerated	Benign	2	AE, SB	1.59E‐04
43128584	rs765317932	c.3202G>A	p.Ala1068Thr	Tolerated	Benign	1	SB	7.98E‐06
Total *PTK7* rare missense variant allele in NTDs compared to gnomAD controls						21/343 versus 4,578/138,632		
						(*p* = 0.00899)		

a Not available.

b AE: anencephaly; CRS: craniorachischisis; EC: encephalocele; EX: Exencephaly; SB: spina bifida.

### 3.2 Functional evaluation of *PTK*7 variants discovered in the NTD cohorts

**Figure 2:**
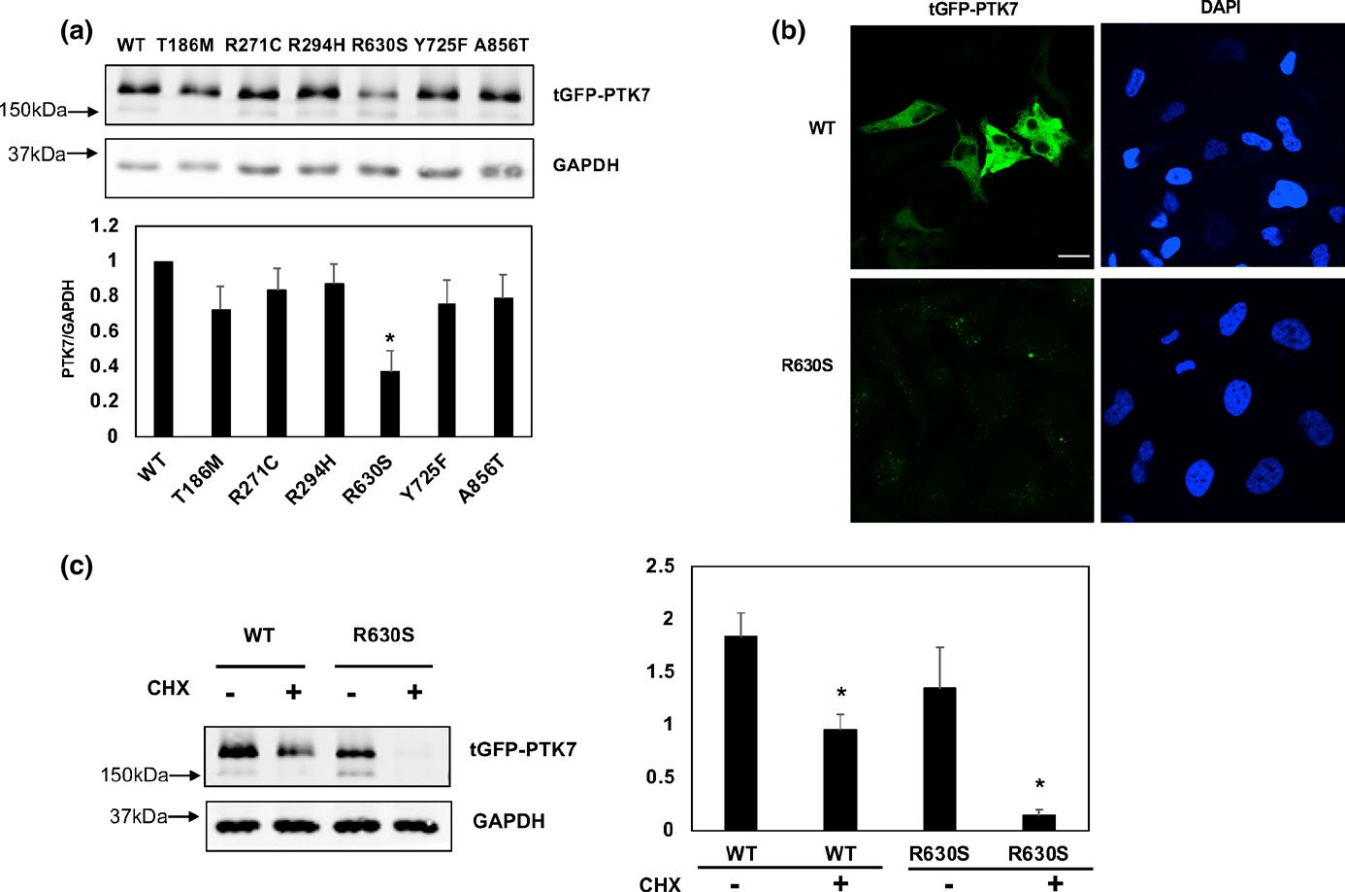
The effect of novel mutations on potentially altering the protein stability. (a) Western blotting assay was performed to detect the expression level of mutated PTK7 constructs with indicated antibodies. Relative expression level which normalized to GAPDH as control, was quantified by Image J. Error bars represent ±*SD* for triplicate experiments. *p < 0.05, Student's *t* test was performed to the wildtype. (b) The protein expression levels of R630S PTK7 and wildtype construct are examined by immunocytochemistry. (c) HEK293T cells were transfected with both R630S and wildtype constructs then treated with CHX at the 1 µg/ml concentrations for 24 hr for blocking protein translation. Error bars represent ±*SD* for triplicate experiments, GAPDH as the control. *p < 0.05 Student's *t* test was performed to the treated and untreated. Scale bar: 20 µm

To validate the effects of three case‐specific missense variants, p.Thr186Met (c.557C>T), p.Arg630Ser (c.1888C>A), and p.Tyr725Phe, we performed western blot analyses to examine protein expression levels. We also performed functional analyses on the three rare missense variants that were discovered only in our control infants. Notably, we found that the p.Arg630Ser variant had significantly lower expression levels compared to the wildtype *PTK7* expression, while the other five missense variants (three from control cohort and two from NTD cohort) maintained almost the same expression levels as did the wildtype allele (Figure 2a). We also performed immunocytochemistry (ICC) assays and found there was a rare signal of GFP (Figure 2b), which supported the result of western blotting assays.

*PTK7* was reported to interact with Wnt genes and inhibit canonical Wnt signaling (Peradziryi et al., 2011). A recent study reported that PTK7 protein level is regulated by its lysosomal degradation (Berger, Breuer et al., 2017). We hypothesized that the reduced expression level of the p.Arg630Ser variant might be secondary to its reduced protein stability, since both lysine and arginine are involved in the ubiquitin dependent protein degradation process. Therefore, we performed protein stability assays by using CHX (cyclohexamide) treatment to block protein translation. Notably, we found that the p.Arg630Ser variant was degraded to about 90% of its total expression after 24 hr of treatment, while wildtype PTK7 was only degraded to about half of its total (Figure 2c). This result indicates that the Arg630 site is crucial to maintain the protein stability of PTK7.

**Figure 3:**
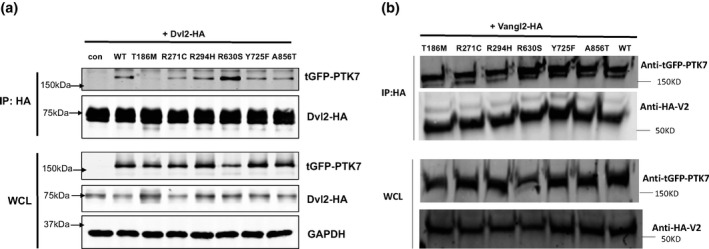
The effect of PTK7 variants on the interaction with Dvl2 and Vangl2. (a) Coimmunoprecipitation assay detecting PTK7‐Dvl2 binding in HEK293T cells by transiently co‐expression of HA‐tagged Dvl2 and tGFP‐tagged PTK7 (wildtype and variants) constructs. (b) Coimmunoprecipitation assay detecting PTK7‐Vangl2 binding in HEK293T cells by transiently co‐expression of HA‐tagged Vangl2 and tGFP‐tagged PTK7 (wildtype and variants) constructs. IP: immunoprecipitation; WCL: whole cell lysate; con: negative control

It is well established that PTK7 recruits Dishevelled protein to regulate neural crest cell migration (Shnitsar & Borchers, 2008). Therefore, we performed co‐immunoprecipitation (Co‐IP) assays to examine the physical interaction between PTK7 proteins and Dvl2 (Dishevelled 2). We found that p.Thr186Met variant affected interaction between PTK7 and Dvl2(Figure 3a), indicating that this site which is located in the second Ig‐like C2‐type domain, contributes to recruiting Dvl2. Three rare variants, which were discovered in the control cohort, together with the p.Tyr725Phe variant, displayed a comparable ability to recruit Dvl2 as did the wildtype protein. Interestingly, the p.Arg630Ser variant showed a much stronger binding ability to Dvl2, compared to wildtype and other variant constructs (Figure 3a).

Previous reports demonstrated that *Ptk7* and *Vangl2* had genetic interactions in mice, and in our Co‐IP assay, we found that GFP‐PTK7 and HA‐Vangl2 protein physically associated with each other. We tested whether the PTK7 variants identified in NTDs could disrupt this physical association. However, none of the tested variants disrupted the interaction between PTK7 and Vangl2 (Figure 3b).

## 4 DISCUSSION

PCP signaling is a key regulator of events involved in epithelial morphogenesis, including NTC, and is mediated by the highly conserved noncanonical Wnt pathway. *Ptk7* is a vertebrate‐specific regulator of PCP. Homozygous *Ptk7* deleterious variants in the mouse disrupted normal NTC, resulting in embryos with craniorachischisis; while fetuses that were doubly heterozygous for *Ptk7* and *Vangl2* presented with spina bifida (Lu et al., 2004). These previous observations led us to explore a potential association between *PTK7* and the risk for human spina bifida. We hypothesized that there would be a significant difference in the number of functional deleterious rare variants between spina bifida cases and non‐malformed controls. In the spina bifida cases, we identified two missense variants that are predicted to be damaging, these variants were not found in the controls or were previously identified in the *ExAC* database. In fact, no novel damaging missense variants were identified in the controls. We also evaluated the association of common *PTK7 *SNPs in spina bifida cases and failed to observe any significant frequency differences for the five common SNPs that were detected. Our results were consistent with the result of previous *PTK7* and NTD study performed by Wang and colleagues (Wang et al., 2015), who identified a single novel damaging/functional missense variant (p.Gly348Ser) in 473 NTD cases, and failed to find any novel damaging missense variants in 150 ethnically matched controls. Although it does not reach statistical significance in their study due to the limitation of sample size. It would require a value of 0 variants in more than 5,000 controls versus 1 in 473 to reach Fisher exact test *p* < 0.05. To validate our findings, we re‐sequenced a different NTD cohort that was also collected in China, and detected 12 predicted‐to‐be damaging rare missense variants. Compared to the EXAC and gnomAD databases, there was a significant (*p* < 0.05) enrichment of predicted‐to‐be damaging rare missense *PTK7* variants in the NTD patients.

PTK7 has been known to interact with several Wnt signaling components, including Wnt ligand, Wnt3a, Wnt8, Wnt4, Wnt5a, and Wnt2, and Wnt receptors such as Fzd1, Fzd2, Fzd7, Ror2, and LRP6, as well as intracellular Wnt components including Dvls and ‐catenin (Reviewed by Hanna Berger et al. [Berger, Wodarz, & Borchers, 2017]). Our functional analyses demonstrated that the residue R630, which is located within the seventh immunoglobulin domain, decreased PTK7 protein stability while it increased its interaction with Dvl2. We also found that the T186M variant changed the amino acid located at the second immunoglobulin domain, thereby decreasing the interaction between PTK7 and Dvl2. Although previous studies found that the kinase homology domain was regulating interactions between PTK7 and Dvls (Shnitsar & Borchers, 2008; Wehner et al., 2011), we found that variants located within the second and seventh immunoglobulin domains could also affect interaction intensity between PTK7 and Dvl2. There could be three possible explanations: the first reason is that variants might affect the PTK7 localization secondary to the dysregulation of endocytosis, the second one is that the PTK7‐Fzd7‐Dvl protein complex might be changed due to mutations at the extracellular domain, the third possibility is that the variant induces a whole conformational change of the protein.

In mice, *Ptk7* heterozygotes did not present with NTDs; while *Ptk7* and *Vangl2^lp^* double heterozygotes had a distinct spina bifida phenotype. To determine whether there are *PTK7* and *VANGL2* combined rare missense variants in human spina bifida cases, we re‐sequenced *VANGL2* in the 192 spina bifida cases used in the current study (data not shown). No novel *VANGL2 *rare missense variants were identified. We also screened for other PCP genes including *CELSR1*(Lei et al., 2014), *SCRIB *(Lei et al., 2013), and *LRP6 *(Lei et al., 2015) in the 192 US spina bifida, but no double PCP damaging missense variants were found in our current analysis. This could be due to the small sample size of our study. Actually, in our recently digenic PCP variants screen in 510 NTDs, *PTK7,* and *SCRIB* double heterozygous variants were identified in a spina bifida case (Wang et al., 2018). Our recently NTD whole genome sequencing study (Chen, Lei, Zheng et al., 2018) indicated that loss of function (LoF) variants in a gene, such as *PTK7*, could interact with other LoF variants in both Wnt pathway and non‐Wnt pathway to increase the genetic risk for NTDs. In the future, large sample size NTDs whole exome and whole genome sequencing study would be able to uncover compound *PTK7* variants and other gene variants which cause NTDs.

The strength of our study is that we included NTDs from two big countries, the USA and China. PTK7 rare variants were identified in NTDs from both cohorts. There are few weaknesses in our study. Firstly, we do not have parent samples to check whether the identified rare *PTK* variants are do novo or not. Secondly, the functional analysis were limited to PCP pathway signaling study. Other non‐PCP/CE functions such as canonical Wnt signaling of PTK7 could be disrupted by variants identified in NTD cases but was not tested in this study.

In conclusion, we detected a total of 18 rare missense variants in human spina bifida. Two of them were functionally validated to affect either protein stability or to compromise the recruitment of Dvl2 protein, which regulates both canonical/non‐canonical Wnt pathways. No novel pathogenic rare variants were identified in non‐malformed controls. Our study indicates that *PTK7* may play a role in the etiology of human NTDs.

## CONFLICT OF INTEREST

The authors have declared that no competing interests exist.
