# Supplementary material for: Variants identified in PTK7 associated with neural tube defects
Source: Mol Genet Genomic Med. 2019 Jan 28;7(4):e00584. doi: 10.1002/mgg3.584 (PMC6465732; doi:10.1002/mgg3.584)
Supplement: Supplementary file 1 [file MGG3-7-na-s001.docx]

**Table S1.** Demographic characteristics in NTD cohorts.

| Variable | China Cohort |
| --- | --- |
| Case # | 343 |
| Age: weeks/years (mean±SD) | |
|  | 22.6±7.0wks |
|  | 5.5±3.9yrs |
| Gender: |  |
| Male | 176 (51.3%) |
| Female | 132 (38.5%) |
| Unknown | 35 (10.2%) |
| AE | 70 (20.4%) |
| CRS | 19 (5.5%) |
| EC | 80 (23.3%) |
| EX | 3 (0.9%) |
| SB | 170 (49.6%) |
| Unknown | 1 (0.3%) |

**AE**, anencephaly; **CRS**, craniorachischisis; **EC**, encephalocele;

**EX**, Exencephaly; **SB**, spina bifida

**Table S2.** Common Variants (MAF>=0.01) in the Coding Sequence of PTK7 Gene Detected in Chinese population.

| Nucleotide change  (NM_002821.3) | dbSNP ID | Amino acid change (NP_002812.2 ) | MAF Control/Case | p Value | gnomad MAF |
| --- | --- | --- | --- | --- | --- |
| c.1851G>A | rs6905948 | p.Gly617Gly | 0.108/0.113 | 0.92 | 0.362 |
